# Editorial: Molecular physiology of smooth muscle cells

**DOI:** 10.3389/fphys.2023.1223278

**Published:** 2023-05-30

**Authors:** Holger Schneider

**Affiliations:** Department of Medicine IV, University Hospital, LMU Munich, Munich, Germany

**Keywords:** TASK-1 channels, l-Type calcium channels (LTCC), dystrophin, intimal hyperplasia (IH), ageing, smooth muscle constriction, rho-associated protein kinase (ROCK), smooth muscle phenotype switch

## Introduction

Our understanding of smooth muscle physiology is continuously changing. Calcium channels were originally considered the main the regulators of smooth muscle tone. Today, we think that smooth muscle tone is modulated by a panoply of ion channels. While voltage-gated potassium channels are an established family of channels for smooth muscle hyperpolarization (Jackson, 2018), the importance of pH-sensitive potassium channels like TASK-1 is just starting to be unravelled.

Myosin light chain (MLC) phosphorylation is the essential determinant of smooth muscle contraction because it increases cross-bridge cycling. MLC phosphorylation state is the result of a Yin-and-Yang-like balance between myosin light chain kinase (MLCK) and myosin light chain phosphatase (MLCP) (Cole and Welsh, 2011). This balance is tipped towards contraction upon depolarization-driven calcium influx through L-type Ca2+ channels. But there is more than just voltage that determines their open probability.

Dystrophin serves as linker between the actin cytoskeleton and the extracellular matrix and likely confers protection to skeletal muscle during contraction (Weller et al., 1990). Dystrophin is also highly expressed in differentiated vascular smooth muscle (Turczyńska et al., 2015), but little is known about its function, especially in the context of germline mutations such as those in the muscular dystrophies.

Vascular injury commonly entails smooth muscle proliferation and phenotype switching (Chakraborty et al., 2021). As the number of involved players like epigenetic regulators increases, an update about the various mechanisms is needed.

Finally, existing evidence suggests that rho-associated kinase (ROK) participates in airway smooth muscle contraction through non-canonical pathways beyond phosphorylation of MLCP (Zhang and Gunst, 2017; Zhang et al., 2018). However, this pleiotropy has not been investigated systematically in the same experimental system. Even less do we know about the differential effects of ageing on ROK-mediated contraction.

This Research Topic within Frontiers in Physiology gives an update about each of the above-mentioned segments of smooth muscle physiology and pathophysiology. The respective contributions were provided by leading scientists in their fields.

### pH-dependent regulation of renal artery tone by acid-sensitive potassium channels

The article by Shvetsova et al. highlights the functional role of TASK-1 channels, pH-sensitive potassium channels, in resistance arteries of the renal and mesenteric circulation. While the contribution of these channels to hypoxic pulmonary vasoconstriction is established (Nagaraj et al., 2013), their functional role in systemic vascular beds was unclear. The group led by Olga Tarasova and Rudolf Schubert demonstrates that TASK-1 channels dampen contractile responses of the renal artery at alkaline extracellular pH values.

These results suggest that under alkaline conditions TASK-1 channels render renovascular smooth muscle less sensitive to vasoconstrictors which results in dilation of the renal artery. One could speculate that the consecutively increased renal blood flow might promote the excretion of HCO_3_^−^ to restore physiological pH.

### Vascular L-type Ca2+ channels—Gating diversity beyond voltage

While classic smooth muscle physiology highlights the activation of L-type Ca2+ channels by depolarization (Nakai et al., 1994), this review by Mironova et al. expands recent insights on cooperative gating by clusters of channels to achieve increased open probabilities which ultimately results in calcium sparklets (Navedo et al., 2005). An additional modulator role is assumed by channel trafficking, the recruiting and de-recruiting of vesicles containing channel molecules to and from the plasma membrane (Ghosh et al., 2018). Putative physiological implications are also presented.

These novel layers of regulation clearly demonstrate that a static concept of L-type Ca2+ calcium channel gating as a simply voltage-driven phenomenon is far from being comprehensive.

### Dystrophin and the dystrophin associated proteins—Uncharted territory in vascular smooth muscle

The next article shifts our focus to an understudied hub linked to actin filaments at the core of which are dystrophin (known through the muscular dystrophies) and the dystrophin associated proteins (DAPs).

Kaplan and Morgan present evidence that dystrophin and the DAPs are expressed in smooth muscle. They subsequently suggest that the skeletal muscular phenotype of muscular dystrophies may also in part be driven by a smooth vascular component. Evidence for this hypothesis stems from human tissue biopsies as well as the mdx mouse model for muscular dystrophy (Miike et al., 1987; Loufrani et al., 2004; Ito et al., 2006).

This review provides an excellent overview about the roles of dystrophin and DAPs in (vascular) smooth muscle and at the same time highlights that a lot of questions remain to be answered.

### Intimal hyperplasia as a model condition for vascular smooth muscle phenotype switch

The article by Déglise et al. focusses on the role of macrovascular smooth muscle in the prevalent clinical problem of intimal hyperplasia following therapeutic interventions in arterial disease. The basic science and its clinical applications are presented back-to-back as a comprehensive state-of-the art review of intimal hyperplasia.

The authors cover the endothelial modulation of the balance between quiescent and proliferative phenotypes as well as the respective pathways which ultimately trigger or repress gene expression programs. As a highlight, the article also outlines the role of diverse non-coding RNA species in the regulation of smooth muscle phenotype. In addition, epigenetic factors such as DNA methylation, histone acetylation and methylation are discussed.

The authors also include a digest of current local treatment modalities such as paclitaxel-coated balloons and sirolimus-eluting stents. They conclude by portraying future therapeutic strategies such as boosting hydrogen sulfate (H_2_S) and inhibiting the YAP/TAZ-TEAD module as effector of the Hippo pathway.

### Ageing and rho-kinase-mediated tone regulation in cerebrovascular smooth muscle

The last article by Lubomirov et al. examines smooth muscle tone in brain arteries as a function of ageing.

The group probes several canonical and non-canonical regulators of smooth muscle contraction to address ageing-specific changes in stretch-induced tone. The authors demonstrate that stretch-induced tone is a feature of senescent basilar arteries (>24 months) in contrast to young (8 months) vessels. They go on to demonstrate a role for non-muscle myosin II specifically in the contraction of senescent arteries, probably via its ROK-regulated polymerization state. Further, they present evidence to suggest that stretch-induced tone in senescent animals is related to decreased inhibition of myosin cross-bridge cycling by caldesmon.

ROK, thus, is shown to mediate ageing-dependent hypercontractility of the basilar artery by multiple mechanisms. Speculating about pathophysiological implications, elder individuals could be more prone to vasospastic ischemia following neurosurgery.

Together, the articles in this Research Topic demonstrate that research on smooth muscle is important for our understanding of health (ion channels, non-canonical ROK-mediated pathways) and disease (muscular dystrophies, intimal hyperplasia).
